# Supplementary material for: Pinging the brain to reveal the hidden attentional priority map using encephalography
Source: Nat Commun. 2023 Aug 7;14:4749. doi: 10.1038/s41467-023-40405-8 (PMC10406833; doi:10.1038/s41467-023-40405-8)
Supplement: Supplementary file 1 — Supplementary Information [file 41467_2023_40405_MOESM1_ESM.pdf]

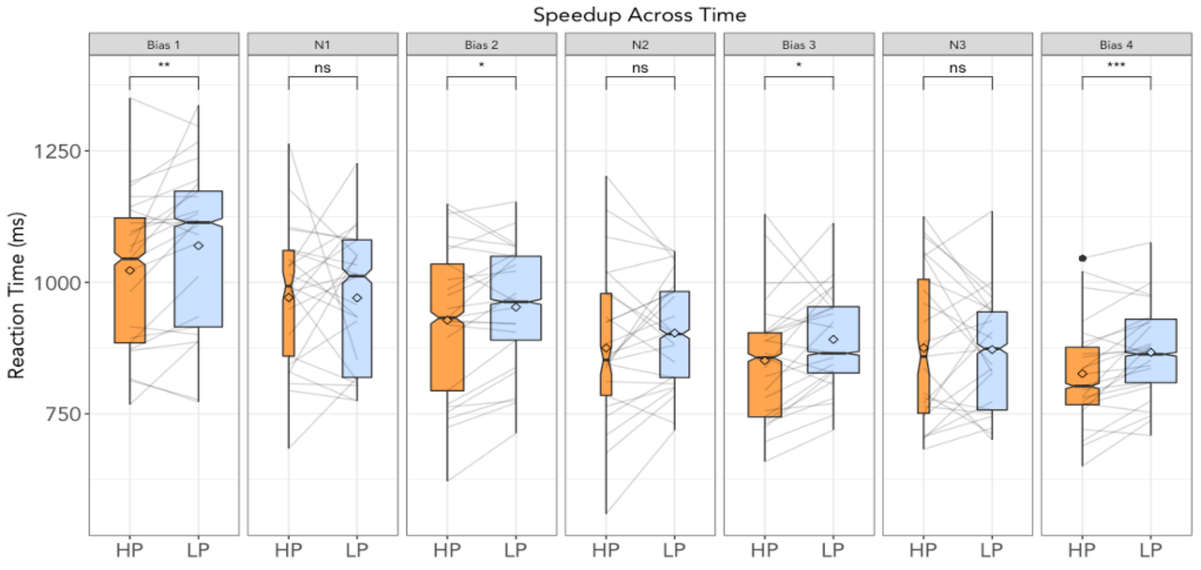

SUPPLEMENTARY FIGURE 1 – Speedup effects across time. RT effects across experiment phases when targets were present at high-probability (orange) versus low-probability (blue) locations in space (see Figure 2 for additional figure nomenclature). Between each set of four bias blocks was a single neutral block (N#). For neutral blocks, there was no HP target location; comparison was thus done using the previous blocks HP location in orange to measure lingering spatial bias from the previous blocks. Note that these averages were calculated on very small trial counts (as illustrated by the narrow bar widths) and so there remains the possibility that an effect exists in these blocks that our analyses were not sensitive enough to detect. Note that each bias phase contained a different high-probability location, counterbalanced across participants. The marked statistics are as follows: Bias 1 (t (23) = 3.485, p = 0.002, d_Z = 0.711, 95% CI [-36.43, -9.291]. N1 (t < 1). Bias 2 (t (23) = 2.283, p = 0.032, d_Z = 0.466, 95% CI [-48.05, -2.368]. N2 (t (23) = 1.031, p = 0.313, 95% CI [-59.58, 19.94]. Bias 3 (t (23) = 2.227, p = 0.36, d_Z = 0.455, 95% CI [-70.86, -2.610]. N3 (t < 1). Bias 4 (t (23) = 3.965, p < 0.001, d_Z = 0.809, 95% CI [-58.59, -18.41].

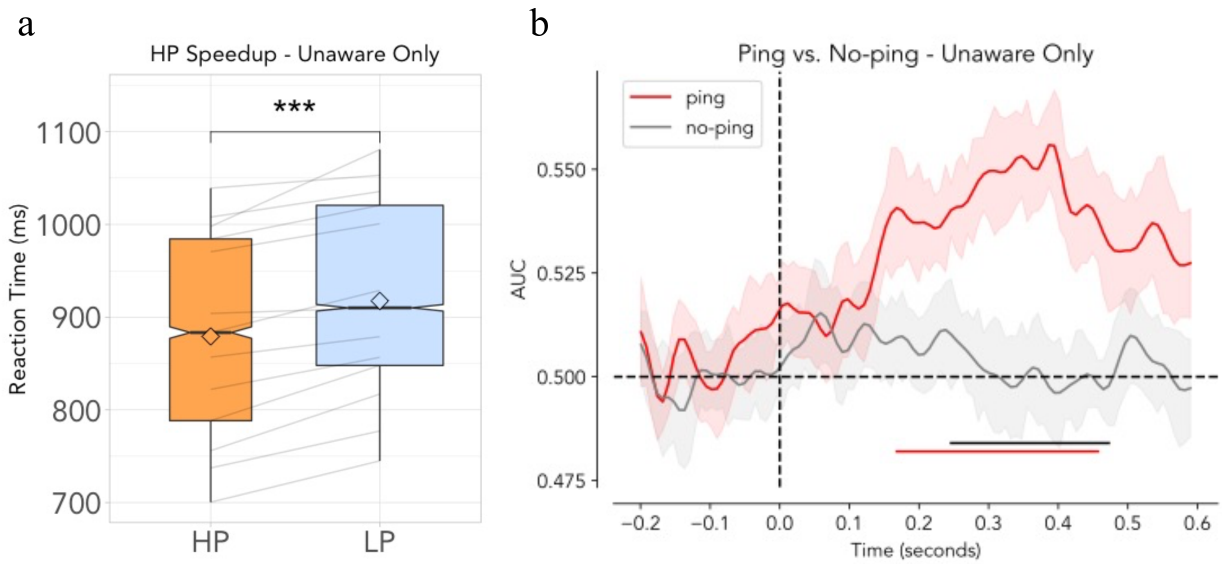

SUPPLEMENTARY FIGURE 2 - Excluding reported-aware participant (N=13). Shown is the behavioral speedup effect (**A**) and decoding results (**B**) of the 13 participants that did not agree in a debrief survey that they noticed some pattern of target spatial distributions. Note that these figures also exclude those who agreed that they notice some pattern of target presentation but were unable to identify where the most recent high-probability location was. Shaded areas in B represent standard error of participant means. See Figure 2 for boxplot nomenclature. *** = p < 0.001.

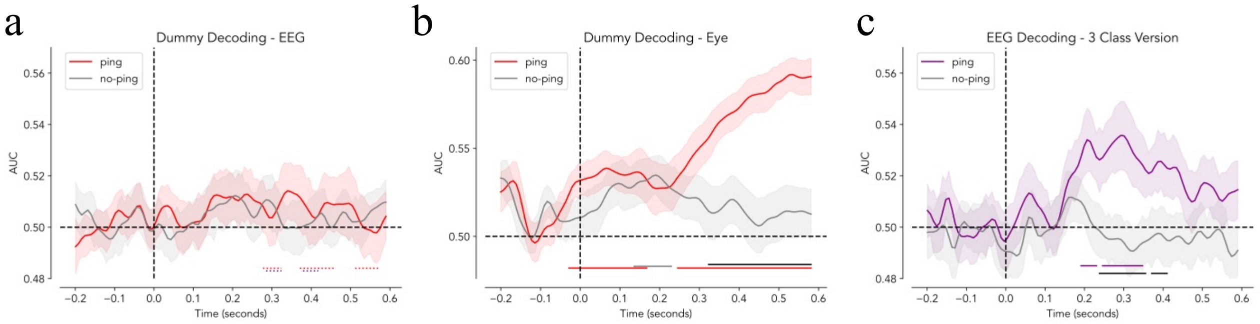

SUPPLEMENTARY FIGURE 3 – Dummy decoding. A) decoder performance when trained on EEG data and passed dummy labels (see figure 1B for illustration of dummy decoding). Dotted red bars indicate clusters in which decoding significantly differed from matched decoding shown in figure 2d. Dotted purple bars indicate the same significant differences but in comparison to supplementary figure 3c. Note: y-axis is matched to results in figure 2d for comparison. B) Decoder performance when trained on eye tracking data and passed dummy labels. C) Hp decoder performance when trained on three classes rather than four; in our decoding results shown in figure 2D, these decoders were tasked with selecting between four classes – each class representing one of the four high-probability target locations. In contrast, our dummy decoders shown in supplementary figure 3A were only tasked with selecting between three classes – fake groups centered around our three neutral blocks. To account for these fundamental differences between these two classification routines, we ran our original decoding on meaningful labels in two iterations – once excluding the first four blocks (the first high-probability conditions), and a second time excluding the last four blocks (the last high-probability condition). These decoders were thus trained on only three classes each, same as the dummy decoding. We then averaged the results of these two iterations to get the results shown here. These results are thus a more valid comparison to our dummy decoding than those shown in figure 2D, and exhibit the same general pattern of results. Shaded areas represent standard error of mean.

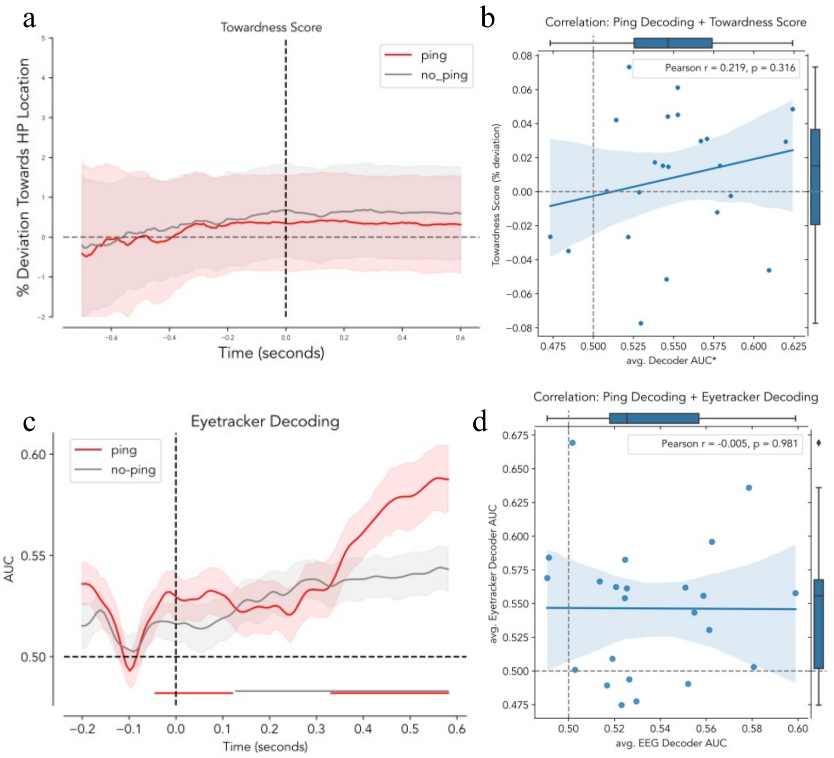

SUPPLEMENTARY FIGURE 4 – Supplementary eye tracking figures (N = 24). A) Towardness scores for each timepoint calculated on un-baselined eye tracker data for the entire intertrial interval (see methods for how eye towardness is calculated). As can be seen, eye position did not change systematically post ping presentation (indicating no systematic microsaccade activity). Additionally, eye position in the un-baselined data did not drift systematically relative to the high-probability location. B) Correlation of individual eye towardness score and decoding accuracy in the window of 300-400ms post ping (this window was chosen as it generally contained the highest decoding). As can be seen, towardness score did not predict decoder accuracy, suggesting eye position did not contribute to decoding. C) Shown is the decoder results when trained on eye tracking data corresponding to the four high-probability locations. Note the similarity of results to those in Supplementary Figure 3B, when the same decoders were trained on meaningless labels. D) Correlation between average decoding for EEG and eye tracking decoders. Shaded areas in decoding figures represent standard error of participant means. Shaded area in scatterplots represent 95% confidence interval of regression estimate. In box plots: shaded box extends over IQR; middle line represents mean; whiskers extend to mini/maximum values; outliers marked as separate dots outside of whiskers if less/more than 1.5 IQR from Q1/3. Significance tests of correlation were two-tailed and unadjusted as they were already non-significant.

a

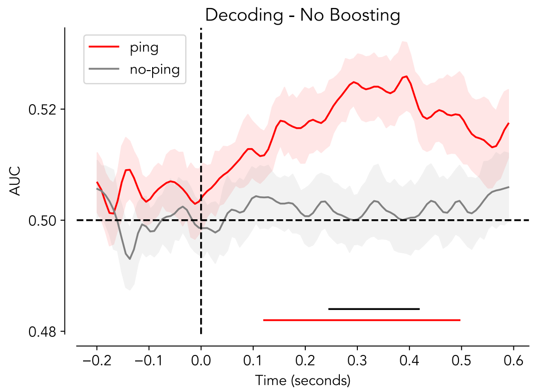

b

Predicted⇒ Actual⇓	Right	Top	Left	Bottom
Right	**28**	*23.6*	*23*	*25.4*
Top	*23.7*	**27.4**	*24.8*	*24.1*
Left	*23.5*	*25*	**27.1**	*24.3*
Bottom	*25.9*	*23.6*	*24.1*	**26.4**

SUPPLEMENTARY FIGURE 5 – Supporting decoding figures. A) In order to increase the signal-to-noise ratio, techniques were borrowed from Grootswagers et al. (2017). These techniques were originally not planned for in the preregistration. The originally planned decoding without these boosting additions, in accord with the preregistered decoding pipeline, are shown in this figure. Note that the general pattern of results does not differ greatly in the timecourse of the overall pattern of decoding from that shown in Figure 2. Shaded areas represent standard error of participant means. B) Shown is the confusion matrix for the boosted decoding shown in figure 2 taken for the period of highest decoding (300-400ms post ping onset. Note that chance accuracy would be 25%). These numbers represent raw decoder accuracy, and thus should be interpreted with caution as they do not linearly translate to different chance baselines (the combined measure of AUC does a better job of representing standardized decoder performance as it combines multiple measurement thresholds).

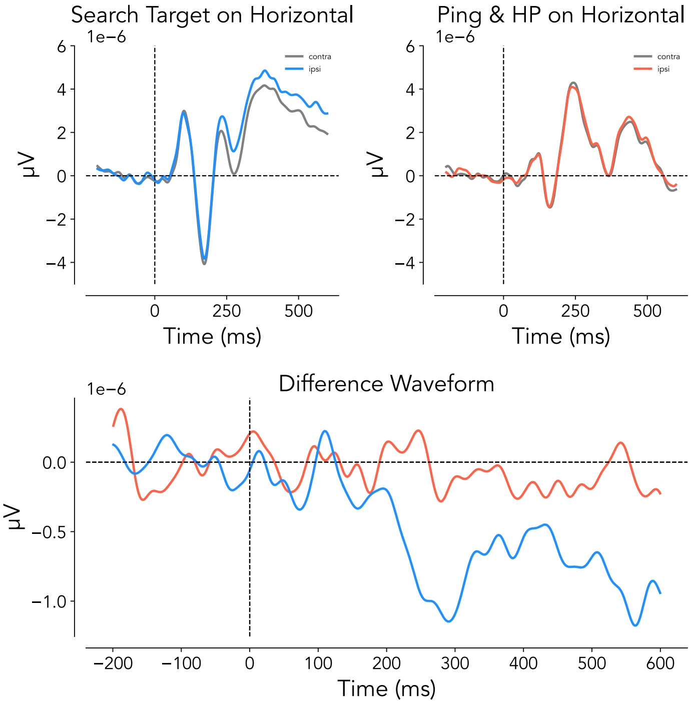

SUPPLEMENTARY FIGURE 6 – Comparing lateralized N2pc evoked components on search trials versus ping trials. Search trials included only trials in which the target appeared on the leftmost or rightmost location in the array – with the evoked response flipped such that the ipsilateral and contralateral side in sensor space were congruent on every trial. Distractors were additionally absent or on the vertical meridian. Ping trials include only trials in which the HP location was on the horizontal midline (left or right) with the same sensor flipping approach used to ensure matched contra/ipsilateral arrangements. As can be see, a clear N2pc was observed in the search trials, but none was observed in the ping trials. These results suggest both that the ping did not evoke a strong attentional response, and also that any decoded results following the ping evoked response cannot be traced back to an evoked N2pc.
