# Supplementary material for: Pinging the brain to reveal the hidden attentional priority map using encephalography
Source: Nat Commun. 2023 Aug 7;14:4749. doi: 10.1038/s41467-023-40405-8 (PMC10406833; doi:10.1038/s41467-023-40405-8)
Supplement: Supplementary file 3 — Reporting Summary [file 41467_2023_40405_MOESM3_ESM.pdf]

nature portfolio

Corresponding author(s): Dock H. Duncan

Last updated by author(s): Jul 6, 2023

Reporting Summary

Nature Portfolio wishes to improve the reproducibility of the work that we publish. This form provides structure for consistency and transparency in reporting. For further information on Nature Portfolio policies, see our Editorial Policies and the Editorial Policy Checklist.

Statistics

For all statistical analyses, confirm that the following items are present in the figure legend, table legend, main text, or Methods section.

n/a	Confirmed	
☐	☒	The exact sample size (*n*) for each experimental group/condition, given as a discrete number and unit of measurement
☐	☒	A statement on whether measurements were taken from distinct samples or whether the same sample was measured repeatedly
☐	☒	The statistical test(s) used AND whether they are one- or two-sided<br>*Only common tests should be described solely by name; describe more complex techniques in the Methods section.*
☐	☒	A description of all covariates tested
☐	☒	A description of any assumptions or corrections, such as tests of normality and adjustment for multiple comparisons
☐	☒	A full description of the statistical parameters including central tendency (e.g. means) or other basic estimates (e.g. regression coefficient) AND variation (e.g. standard deviation) or associated estimates of uncertainty (e.g. confidence intervals)
☐	☒	For null hypothesis testing, the test statistic (e.g. *F*, *t*, *r*) with confidence intervals, effect sizes, degrees of freedom and *P* value noted<br>*Give P values as exact values whenever suitable.*
☐	☒	For Bayesian analysis, information on the choice of priors and Markov chain Monte Carlo settings
☒	☐	For hierarchical and complex designs, identification of the appropriate level for tests and full reporting of outcomes
☒	☐	Estimates of effect sizes (e.g. Cohen's *d*, Pearson's *r*), indicating how they were calculated

Our web collection on statistics for biologists contains articles on many of the points above.

Software and code

Policy information about availability of computer code

Data collection	custom scripts were used in the OpenSesame experimental framework (version 3.3). EEG data was collected using a BioSemi set and recorded into ActiView (v. 7.07). Eyetracking was collected using several Eyelink 1000's (several versions used) with data recorded in the SR research host software (v 4.594)
Data analysis	EEG and eyetracking data analysis was done using custom Python code (https://github.com/dvanmoorselaar/DvM) making heavy use of the MNE framework (v. 1.1.1). Behavioral data was analyzed in R (v 4.2.1). Bayesian analyses were conducted using JASP (v 0.16.1). Custom python script can be found at (https://osf.io/v7yhc/).

For manuscripts utilizing custom algorithms or software that are central to the research but not yet described in published literature, software must be made available to editors and reviewers. We strongly encourage code deposition in a community repository (e.g. GitHub). See the Nature Portfolio guidelines for submitting code & software for further information.

Data

Policy information about availability of data

All manuscripts must include a data availability statement. This statement should provide the following information, where applicable:

- Accession codes, unique identifiers, or web links for publicly available datasets
- A description of any restrictions on data availability
- For clinical datasets or third party data, please ensure that the statement adheres to our policy

All scripts related to EEG preprocessing and analysis, behavioral data preprocessing and analysis, as well as the experiment code will be made available on the

project's OSF page (https://osf.io/v7yhc/) and the associated toolbox's Github (https://github.com/dvanmoorselaar/DvM).

Research involving human participants, their data, or biological material

Policy information about studies with human participants or human data. See also policy information about sex, gender (identity/presentation), and sexual orientation and race, ethnicity and racism.

Reporting on sex and gender	no sex- or gender- based analyses were done. number of male/female reporting participants is included.
Reporting on race, ethnicity, or other socially relevant groupings	no further race, ethnicity, or other social variables were collected
Population characteristics	24 participants. 17 reported sex female, 7 reported male. mean age 24
Recruitment	participants were recruited via the VU Amsterdam participant recruitment system (SONA). Participants were primarily bachelor students in the Psychology program who participated for course credits or money. Participants were self selected volunteers from a northern European country with access to a university education, and were thus not a representative sample of the overall population of the Earth (as is common in cognitive psychology research). There is no a priori reason to suspect this population drastically differed in the low-level cognitive mechanisms presently under study.
Ethics oversight	Ethical Review Committee of the Faculty of Behavoural and Movement Sciences, Vrije Universiteit Amsterdam

Note that full information on the approval of the study protocol must also be provided in the manuscript.

Field-specific reporting

Please select the one below that is the best fit for your research. If you are not sure, read the appropriate sections before making your selection.

☒ Life sciences ☐ Behavioural & social sciences ☐ Ecological, evolutionary & environmental sciences

For a reference copy of the document with all sections, see nature.com/documents/nr-reporting-summary-flat.pdf

Life sciences study design

All studies must disclose on these points even when the disclosure is negative.

Sample size	Sample sizes were based on those used in other similar experimental designs (Wolff et. al., 2015, 2017, 2020 & 2021). final sample size of 24 participants was decided to satisfy condition-order counterbalancing. No statistical method was used to predetermine sample size
Data exclusions	pre-established data exclusion criteria necessitated the replacement of 8 participants in our final dataset. 2 participants were excluded for poor EEG quality; 2 for consistent failures to maintain fixation as shown from the eyetracking and EOG data; 2 participants were excluded for having reaction times 2.5 standard deviations above the group mean; and 2 participants were excluded for having final task accuracies 2.5 standard deviations below the group average.
Replication	no replication was undertaken, as is normal in experiments using cross-validated (10 fold) decoding analyses
Randomization	condition counterbalancing was done. in the analysis, decoders were trained using a randomized cross-validation technique with ten folds
Blinding	blinding was not relevant to study as it was a within-subject design with no separate testing/control populations. Participants were naive to the experiments purpose.

Reporting for specific materials, systems and methods

We require information from authors about some types of materials, experimental systems and methods used in many studies. Here, indicate whether each material, system or method listed is relevant to your study. If you are not sure if a list item applies to your research, read the appropriate section before selecting a response.

Materials & experimental systems

n/a	Involved in the study	
☒	☐	Antibodies
☒	☐	Eukaryotic cell lines
☒	☐	Palaeontology and archaeology
☒	☐	Animals and other organisms
☒	☐	Clinical data
☒	☐	Dual use research of concern
☒	☐	Plants

Methods

n/a	Involved in the study	
☒	☐	ChIP-seq
☒	☐	Flow cytometry
☒	☐	MRI-based neuroimaging
